# Successful treatment of lichen spinulosis with topical roflumilast: A case report

**DOI:** 10.1177/2050313X251400983

**Published:** 2025-12-04

**Authors:** Rose He, Yitong Xu, Karen Naert, Justin C. Chia

**Affiliations:** 1 Cumming School of Medicine, University of Calgary, AB, Canada; 2 Department of Pathology and Laboratory Medicine, University of Calgary, AB, Canada; 3 Division of Dermatology, University of Calgary, AB, Canada

**Keywords:** roflumilast, lichen spinulosis, PDE4 inhibitor

## Abstract

Lichen spinulosis is an uncommon, follicular keratotic disorder characterized by asymptomatic clusters of follicular papules with keratotic spines that primarily occurs in children, and rarely in adults. Various treatment options have been reported in the literature, although none are universally effective. Topical roflumilast (0.3%) is a recently approved phosphodiesterase-4 inhibitor first used in psoriasis which has shown reduction in hyperkeratosis in vitro. We report on an adult with symptomatic and recalcitrant lichen spinulosis who achieved significant improvement with the use of topical roflumilast over a 6-month period.

## Introduction

Lichen spinulosis (LS) is a member of the family of follicular keratotic disorders that is characterized by asymptomatic clusters of follicular papules with keratotic spines, predominantly distributed on the trunk and extremities. The pathogenesis involves plugging of the hair follicle by follicular hyperkeratosis, with the etiology largely unknown, although associations with atopy have been suggested.^1^ It mainly affects children and young adults, with rare occurrences in adults. Treatments for symptomatic and persistent LS commonly include keratolytics (e.g., 3%–6% salicylic acid, 10%–40% urea, ammonium lactate), emollients, mid-potency corticosteroids, vitamin D analogues (e.g., calcipitriol) and retinoids (e.g., tretinoin gel, adapalene) with increased efficacy under occlusion.^1–5^ No treatments have been universally effective, and chronic usage of topical steroids can result in epidermal atrophy.

Roflumilast cream (0.3%) is a novel, nonsteroidal cream, that has demonstrated safety and efficacy for use in plaque psoriasis, seborrheic dermatitis, and mild-to-moderate atopic dermatitis by inhibiting phoshodiesterase 4 (PDE4).^6,7^ PDE4 inhibitors have been shown to reduce hyperproliferation of keratinocytes in human skin,^8^ and, therefore, could potentially aid in the treatment of follicular keratotic disorders. In this case report, we present an adult patient with recalcitrant LS who was successfully treated with topical roflumilast, a PDE4 inhibitor.

## Case

**Figure 1.**
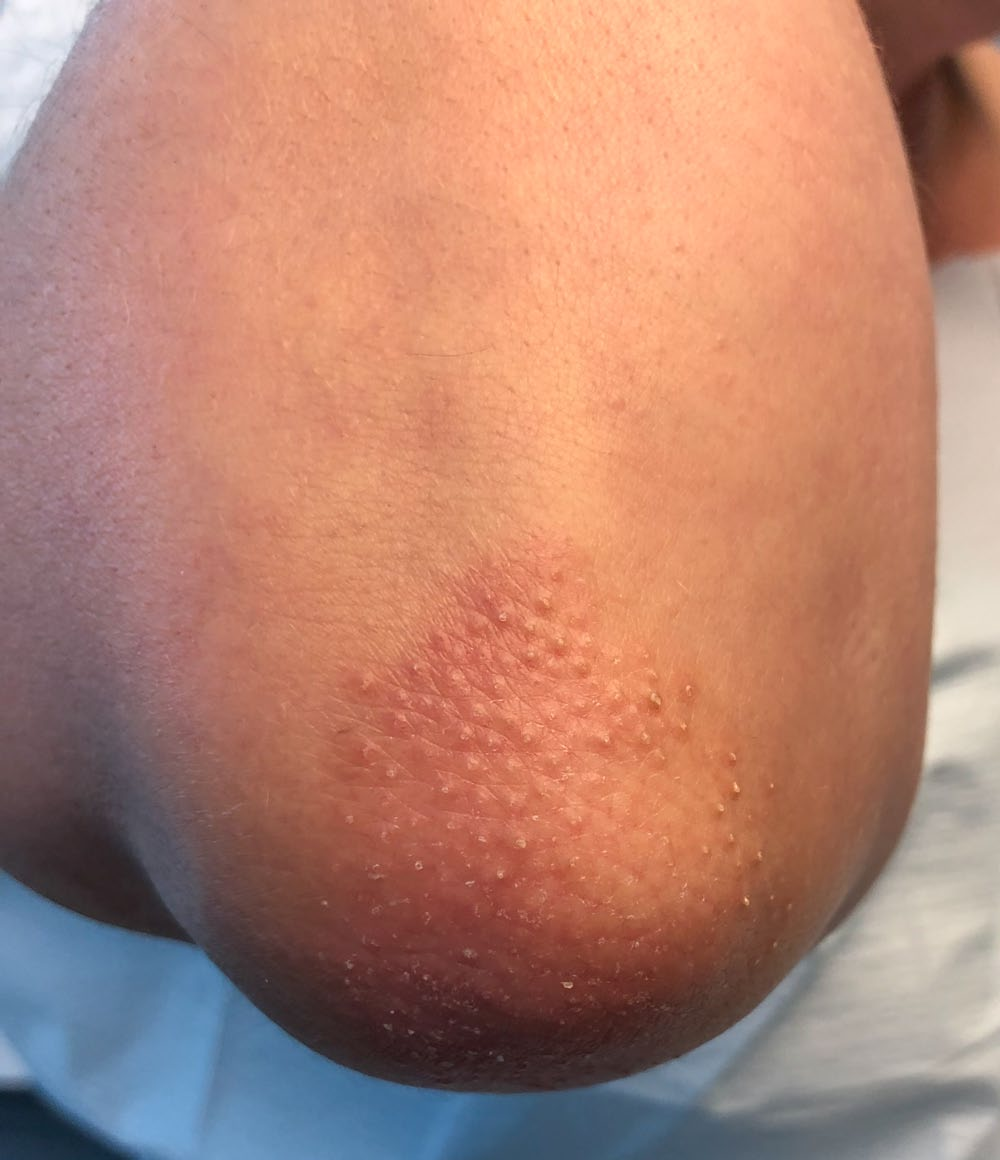
Clinical appearance of the untreated right elbow with follicular hyperkeratotic spines.

**Figure 2.**
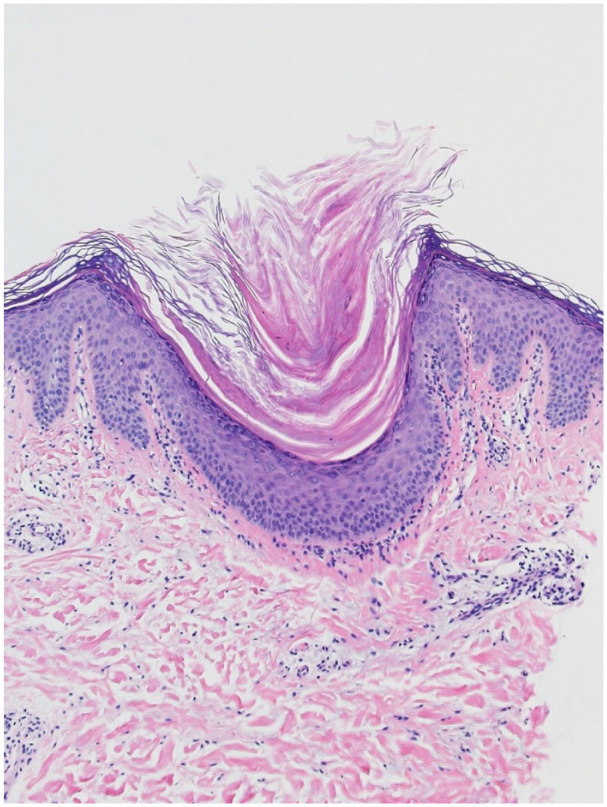
Representative histological (hematoxylin and eosin) image of the right elbow biopsy. Note the prominent keratinous follicular plugging with a sparse perifollicular lymphocytic infiltrate.

**Figure 3.**
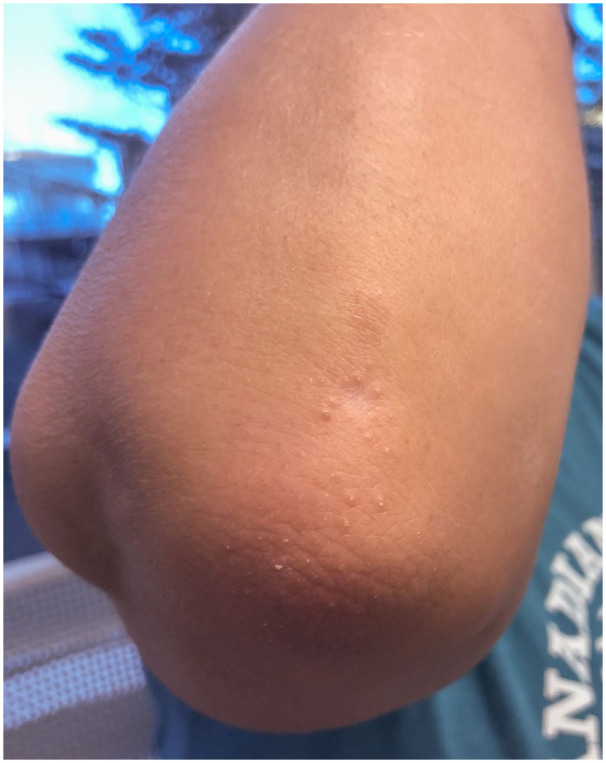
Clinical appearance of the treated right elbow after 6 months of topical roflumilast.

A 51-year-old female presented to our dermatology clinic with a 3-year history of follicular spines on the elbows bilaterally (Figure 1). She found the spines could be tender when she rested her elbows on a desk. Potent topical corticosteroids were trialed in the past with minimal efficacy. A skin biopsy was performed and showed prominent keratinous follicular plugging with a mild perifollicular lymphocytic infiltrate, consistent with LS (Figure 2). The patient was initiated on calcipitriol (50 μg/g)/betamethasone dipropionate (0.5 mg/g) gel with some improvement within 1 month, but the hyperkeratosis recurred at 2 months despite continued treatment. She then trialed halobetasol (0.01% w/w)/tazarotene (0.045% w/w) for 3 months with no further improvement. All treatment was stopped for the following 3 years due to minimal efficacy. The patient was later re-referred to our clinic, and topical roflumilast (0.3%) was then initiated once daily, and at 5 weeks of treatment, she achieved 50% improvement and 95% improvement at 6 months (Figure 3). There were no reported side effects and she remained on the same treatment with continued efficacy.

## Discussion

Lichen spinulosus is a condition of follicular hyperkeratosis which has few effective treatments. It can greatly impact the quality of life of patients, particularly when it occurs on the elbows and buttock. This case illustrates the use of topical roflumilast cream in LS after failure of other treatment options.

Roflumilast cream is a potent and selective PDE4 inhibitor, modulating cyclic adenosine monophosphate levels to reduce pro-inflammatory cytokines and thereby lessening inflammation and normalizing keratinocyte proliferation.^7^ We propose that this normalizing of keratinocyte proliferation is what gives it efficacy in LS. Roflumilast is approximately 25–300-fold more potent than oral apremilast and topical crisaborole in vitro, which are the two other PDE4 inhibitors approved for dermatologic conditions.^7^ Roflumilast cream (0.15%) has been approved for those age 6 and over for atopic dermatitis; roflumilast (0.3%) cream is approved for those 12 and older for plaque psoriasis; and roflumilast foam (0.3%) has been approved for age 9 and over for seborrheic dermatitis. Given that LS predominately occurs in the pediatric population, it is a promising option in this age group as it can be used long-term with minimal side effects. No studies to date have been done using topical roflumilast in other disorders of follicular hyperkeratosis, but the authors suspect it may also work in similar conditions.

## Conclusion

We report success in utilizing roflumilast cream (0.3%) as a monotherapy for treatment of recalcitrant LS in an adult, with ongoing efficacy. Further studies should be done to determine efficacy in a larger number of patients, especially in the pediatric population. We would encourage practitioners to consider topical roflumilast cream (0.3%) as a first-line agent in LS given its tolerability, safety, and durability.
